# Pharmacotherapy of Neuropathic Pain

**DOI:** 10.3390/ph18060930

**Published:** 2025-06-19

**Authors:** Sergio Marques Borghi

**Affiliations:** 1 Laboratory of Pain, Inflammation, Neuropathy, and Cancer, Department of Pathology, Londrina State University, Londrina 86057-970, PR, Brazil; sergio_borghi@yahoo.com.br or sergio.borghi@cogna.com.br; 2 Center for Research in Health Sciences, University of Northern Paraná, Londrina 86041-140, PR, Brazil

Despite advances in recent decades, the management of neuropathic pain remains one of the greatest challenges in modern clinical medicine. Neuropathic pain decreases patients’ quality of life, leading to major social, economic, and psychological losses, and, importantly, it represents an undesirable economic burden on society when considering the financial costs of its chronic treatment, especially for individuals with moderate to severe pain [1,2,3]. The physiopathology of neuropathic pain can vary significantly depending on the disease with which it is associated. Damage to the nervous system, including the peripheral nociceptor, spinal cord, and cortical brain, may precipitate neuropathic pain, demonstrating its immense complexity [4] and highlighting the necessity of identifying successful research and new pharmacotherapeutic and non-pharmacotherapeutic interventions for its treatment [2]. In this sense, both preclinical studies and clinical trials are essential to achieve this goal.

Focusing on developing new solutions or methods by using comprehensive strategies can further advance our understanding of neuropathic pain management. Studies aimed at discovering potential new molecular and/or cellular pharmacological targets, as well as drug repurposing, combination therapy, innovative nanotechnology, gene therapies, non-pharmacological approaches such as axon therapy and neuromodulation, and precision medicine (personalized therapeutic medical assistance), are examples of interesting options suitable for further investigation [2,5]. This Special Issue of *Pharmaceuticals*, “Pharmacotherapy of neuropathic pain”, comprises eleven original research articles and four review articles that address new research on these topics. The findings of these studies are briefly discussed below.

Using a murine chronic constriction injury (CCI) model, Zajączkowska and colleagues demonstrated that the gabapentinoid mirogabalin’s analgesic effect is superior to that of pregabalin. They also revealed that its mechanistic effects are related to the prevention of glial cell/macrophage activation and neutrophil infiltration through the inhibition of p38 mitogen activated protein (MAP) kinase and pronociceptive chemokines [Contribution 1]. In another original article, by employing a rat partial sciatic nerve ligation (pSNL) model, it was found that pregabalin/tolperisone combination therapy effectively reduces neuropathic pain and, although not in a significant fashion, attenuated the cerebrospinal fluid levels of the predominant excitatory neurotransmitter glutamate in the dorsal horn of the spinal cord [Contribution 2]. A pSNL rat model was also used to explore the role of the melatonin type 2 (MT2) receptor in morphine analgesic tolerance in regard to neuropathic pain. MT2 agonist IIK7 treatment (administered intrathecally) delayed or reversed morphine analgesic tolerance, depending on the dose tested. The authors attributed these effects to a molecular mechanism related to the induction of nuclear factor erythroid 2-related factor 2 (Nrf2) transcription factor and its downstream effector heme-oxygenase -1 (HO-1) alongside to the inhibition of both glial cell activity and the production of pro-inflammatory cytokines such as tumor necrosis factor (TNF)-α, interleukin (IL)-1β, and IL-6 [Contribution 3], highlighting IIK7’s potential as a candidate for clinical trials aimed at delaying and/or reversing morphine analgesic tolerance during clinical neuropathies. Platinum-based antineoplastic drugs are known to induce chemotherapy-induced peripheral neuropathy [6]. Gang and colleagues used a murine model of oxaliplatin-induced peripheral neuropathy (OIPN) to investigate the phenolic compound [6]-Shogaol extracted from ginger rhizomes. They found that [6]-Shogaol can attenuate allodynia by increasing both serotonin-synthesizing enzyme tryptophan hydroxylase 2 (TPH2) levels and the levels of serotonin itself acting through 5-HT_1A_ and 5-HT_3_ receptors in the spinal serotoninergic system, thus unraveling an important mechanism whereby this compound can induce analgesia in chemotherapy-induced peripheral neuropathy [Contribution 4].

Cannabigerol (CBG) is a non-psychoactive cannabinoid and minor constituent of cannabis with analgesic potential [7]. One article in this Special Issue demonstrates that CBG may act as a long-lasting analgesic, as shown in a murine cisplatin-induced peripheral neuropathy (CIPN) model, without inducing tolerance, weight change, or adverse events in animals [Contribution 5]. Trigeminal neuralgia is a common form of neuropathic pain. Using a chronic a rat constriction injury model of the infraorbital nerve (IoN-CCI), a group of authors showed that URB937, a blood–brain-barrier-impermeant fatty acid amide hydrolase (FAAH) (the degrading enzyme of the endocannabinoid anandamine) inhibitor, may function as a preventive but not therapeutic treatment for IoN-CCI -induced mechanical allodynia. These effects were attributed to the inhibition of hyperalgesic molecules such as calcitonin gene-related peptide (CGRP), IL-1β, and TNF-α and the induction of the activity of the anti-hyperalgesic cytokine IL-10 in tissues like the medulla, cervical spinal cord, and trigeminal ganglion [Contribution 6]. As URB937 showed preventive effects, this preclinical evidence is critically important; although the findings have not been tested in a model of neuropathy caused by chemotherapy, they may represent potential treatment options for preventing pain in patients undergoing such treatment, providing an interesting hypothesis to investigate further.

Fructose-1,6-bisphosphate (FBP) is an intermediate product of the glycolytic pathway. It was previously demonstrated that FBP can have analgesic effects on inflammatory pain by increasing blood adenosine production and signaling through the adenosine 1A receptor (A1AR) [8]. Therefore, our group investigated the effects of FBP on neuropathic pain using a murine sciatic nerve CCI model. FBP inhibited CCI-induced mechanical hyperalgesia, and both the A1A and A2A receptors mediated its analgesic effects. It was shown that FBP’s mechanistic action depends on increasing nitric oxide (NO) production in dorsal ganglia (DRG) neurons, activating the NO/cyclic guanosine monophosphate (cGMP)/protein kinase G (PKG)/adenosine triphosphate (ATP)-sensitive potassium channel (KATP) signaling pathway, and reducing TRPA1^+^ DRG neuron activity to induce analgesia [Contribution 7]. Rapacz and colleagues studied the antiseizure and analgesic properties of 3-(benzo[b]thiophen-2-yl)pyrrolidine-2,5-dione derivatives. Among the compounds identified, one dubbed compound 33 was considered the most promising. Compound 33 efficiently reduced allodynia in an OIPN murine model, with the proposed mechanism of action involving modulatory activity effects on neuronal voltage-sensitive sodium channels. Importantly, in vitro data showed no hepatotoxicity, neurotoxicity, or mutagenic effects, highlighting the safety of this compound [Contribution 8].

The Special Issue also presents clinical trials. In a multicenter, randomized, placebo-controlled, four-arm parallel trial, cannabis-based medicine was tested in patients with multiple sclerosis and spinal cord injuries presenting neuropathic pain. Different from what was previously observed in a CCI model of neuropathic pain in mice (i.e., pain reduction) [9], treatments with delta-9-tetrahydrocannabinol (THC), cannabidiol (CBD), or a combination of THC/CBD for 6 weeks were not superior to a placebo in terms of the intensity of patient-reported neuropathic pain. Spasticity intensity and the patient global impression of change and quality of life also remained unchanged [Contribution 9]. A new potential treatment for patients with diabetic polyneuropathy has also been tested. α-lipoic acid is used for the management of chronic diseases characterized by oxidative stress, including diabetic neuropathy [10]. In a pre/post experimental design, significant reductions in pain intensity and plasmatic concentrations of biomarker of DNA damage 8-hydroxy-2′-deoxyguanosine (8-OHdG) in patients with diabetic polyneuropathy were observed after short-term intravenous treatment with α-lipoic acid. These results are very interesting; however, they should be confirmed in future studies via randomized controlled trials [Contribution 10]. Lastly, in their retrospective study, Kopsky et al. showed that the topical use of phenytoin (a drug applied for the management and treatment of epilepsy) cream promotes, without systemic side effects, prolonged pain relief after extended use when compared to a placebo among patients with painful diabetic neuropathy [Contribution 11].

Regarding review articles, the topics covered include a narrative review on central post-stroke pain (CPSP), a narrative review on the effects of topical pharmacotherapy for intra-oral post traumatic trigeminal neuropathic pain (IOPTTNP), a scoping review on potential cholinergic compounds useful for peripheral neuropathic pain management based on preclinical studies, and a narrative review providing a general overview of preclinical models and medications for peripheral neuropathy. Mohanan et al. brought to light that antidepressants such as amitriptyline and lamotrigine are the first-line medications for CPSP, while gabapentinoids constitute the second-line options. Additionally, the combination of antidepressants with gabapentinoids may be more effective with reduced drug doses, and non-pharmacological choices may be an option for cases non-responsive to pharmacotherapy [Contribution 12]. Sharav and colleagues conducted searches of current data on the effects of topical therapy for IOPTTNP. The authors concluded that these types of interventions may offer targeted pain-relief with minimal adverse effects and that these effects can be improved when a range of different analgesic agents are incorporated, suggesting a benefit in utilizing a diverse array of pain-relieving strategies. However, they also highlight that due to the paucity of available data and the methodological limitations of the corresponding studies, further investigations using clinical trials with standardized procedures and long-term follow-ups are essential to validate the efficacy of these treatments [Contribution 13]. Montigné and Balayssac found that muscarinic and nicotinic acetylcholine receptor (mAChER and nAChER, respectively) ligands remain the most investigated targets. Examples include α4β2 and α7 nAChR agonists and α9/α10 nAChR antagonists. For mAChRs, no distinct cholinergic target was proposed. The authors conclude that based on preclinical data, cholinergic compounds have great potential for the management of peripheral neuropathicpain and therefore deserve to be further evaluated in future clinical studies [Contribution 14]. The narrative review by Jali et al. underscores the advantages and limitations of the main models of peripheral neuropathy applied in preclinical studies and advocates an integrated approach to addressing the complexities of its pathophysiology, which, according to the authors, would make it possible to optimize treatment results [Contribution 15].

In conclusion, this Special Issue provides significant new studies contributing to expanding the possibilities of new pharmacological investigations on neuropathic pain. However, this topic still has many critical questions that remain unanswered, promising a challenging future for this field. I sincerely thank all the authors and reviewers for their valuable contributions to this Special Issue.
